# Immunogenicity and safety of SARS-CoV-2 vaccine in hemodialysis patients: A systematic review and meta-analysis

**DOI:** 10.3389/fpubh.2022.951096

**Published:** 2022-09-23

**Authors:** Ren Peiyao, Yu Mengjie, Shen Xiaogang, He Wenfang, Zheng Danna, Zeng Yuqun, Jin Juan, He Qiang

**Affiliations:** ^1^Second Clinical Medical School, Zhejiang Chinese Medical University, Hangzhou, China; ^2^Urology and Nephrology Center, Department of Nephrology, Zhejiang Provincial People's Hospital, Affiliated People's Hospital, Hangzhou Medical College, Hangzhou, China; ^3^Department of Nephrology, The First Affiliated Hospital of Zhejiang Chinese Medical University (Zhejiang Provincial Hospital of Traditional Chinese Medicine), Hangzhou, China

**Keywords:** COVID-19 vaccine, dialysis, immunogenicity, end stage kidney disease (ESKD), system review, meta-analysis

## Abstract

**Rationale and objective:**

COVID-19 vaccination is the most effective way to prevent COVID-19. For chronic kidney disease patients on long-term dialysis, there is a lack of evidence on the pros and cons of COVID-19 vaccination. This study was conducted to investigate the immunogenicity and safety of COVID-19 vaccines in patients on dialysis.

**Methods:**

PubMed, MEDLINE, EMBASE, and the Cochrane Library were systemically searched for cohort, randomized controlled trials (RCTs), and cross-sectional studies. Data on immunogenicity rate, antibody titer, survival rate, new infection rate, adverse events, type of vaccine, and patient characteristics such as age, sex, dialysis vintage, immunosuppression rate, and prevalence of diabetes were extracted and analyzed using REVMAN 5.4 and Stata software. A random effects meta-analysis was used to perform the study.

**Results:**

We screened 191 records and included 38 studies regarding 5,628 participants. The overall immunogenicity of dialysis patients was 87% (95% CI, 84-89%). The vaccine response rate was 85.1 in hemodialysis patients (HDPs) (1,201 of 1,412) and 97.4% in healthy controls (862 of 885). The serological positivity rate was 82.9% (777 of 937) in infection-naive individuals and 98.4% (570 of 579) in patients with previous infection. The Standard Mean Difference (SMD) of antibody titers in dialysis patients with or without previous COVID-19 infection was 1.14 (95% CI, 0.68–1.61). Subgroup analysis showed that the immunosuppression rate was an influential factor affecting the immunogenicity rate (*P* < 0.0001). Nine studies reported safety indices, among which four local adverse events and seven system adverse events were documented.

**Conclusions:**

Vaccination helped dialysis patients achieve effective humoral immunity, with an overall immune efficiency of 87.5%. Dialysis patients may experience various adverse events after vaccination; however, the incidence of malignant events is very low, and no reports of death or acute renal failure after vaccination are available, indicating that vaccine regimens may be necessary.

**Systematic review registration:**

https://www.crd.york.ac.uk/PROSPERO/display_record.asp?ID=CRD42022342565, identifier: CRD42022342565.

## Introduction

Since the rapid transmission and wide variability of the novel coronavirus, developing a highly effective vaccine against the stubborn pathogen has become vital (1). Several SARS-CoV-2 vaccines have been developed and are currently administered to people worldwide to achieve effective immunity (2). According to a cohort study in Chile involving 10.2 million people, inactivated vaccines were effective at preventing COVID-19 as well as reducing the incidence of severe disease and death (3). The latest clinical trials have demonstrated that they can effectively reduce morbidity and mortality and the incidence of adverse events in a healthy population (4, 5). Vaccination against COVID-19 raises the hope that humans can defeat the disease.

Patients with end-stage renal disease (ESRD) rely on hemodialysis (HD), peritoneal dialysis (PD), and other renal replacement therapies to facilitate the removal of toxins and metabolic waste from the body to compensate for a patient's dysfunctional kidneys and maintain the body's water and acid-base balance. Multiple complications are often associated with dialysis, of which diabetes mellitus and hypertension are the most closely related (6). Additionally, advanced age, diabetes, hypertension, and smoking are all risk factors for COVID-19 (7, 8). Furthermore, the long-term use of immunosuppressants and the loss of immune proteins caused by the increase in renal basement membrane permeability jointly led to immunosuppression in dialysis patients. In such situations, HDPs were at a higher more at risk of COVID-19 infection, and may lead to adverse outcomes (9). Therefore, it can be assumed that dialysis patients benefit from an effective vaccine. However, for immunocompromised patients, inadequate immune efficacy after other vaccination such as hepatitis B vaccine has raised concern of the efficacy and safety of COVID-19 vaccines (10, 11). Currently, the benefits and costs of COVID-19 vaccination for immunocompromised populations still remain controversial.

Given the higher infection rate and lower resistance to virus than healthy individuals, the risks of vaccination in HDPs should be considered (12). After all, it remains to be seen whether patients with an immune deficiency can produce an adequate immune response against the virus. Furthermore, patients with impaired immunity risk experiencing uninformed health problems due to the toxicity of the vaccine itself. Therefore, more convincing evidence regarding the efficacy and safety of COVID-19 vaccines in hemodialysis patients is needed. This study was aimed to summarize available evidence on the efficacy and safety of COVID-19 vaccines in HDPs and to guide clinical practice.

## Methods

A systematic review and meta-analysis were performed strictly per the Preferred Reporting Items for Systematic Reviews and Meta-Analyses (PRISMA). This meta-analysis has been recorded in the International Prospective Register of Systematic Reviews (PROSPERO) database (ID: CRD42022342565).

### Search strategy

PubMed, MEDLINE, EMBASE, and Cochrane Library databases were searched for relevant articles published between January 1st, 2020 and September 30th, 2021, with medical subject headings (MeSH) terms and the corresponding entry terms. Additional search details can be found in the Supplementary materials. To conduct a comprehensive search, the references listed in the retrieved studies were reviewed for comparison.

### Study selection

Prospective cohort studies, randomized controlled trials (RCTs), and cross-sectional studies investigating the immunogenicity of COVID-19 vaccines in patients undergoing maintenance hemodialysis were included. Studies reporting adverse events and vaccine safety were also included. Studies that reported immunogenicity only in peritoneal dialysis patients and kidney transplant recipients and non-English studies were excluded. Reference management software, Endnote, was used to find and remove duplicate literatures.

### Data extraction

As part of the data extraction procedure, the literature was independently screened, and the included studies' titles, abstracts, and full text were checked. Patient characteristics, such as age, sex, rate of previous immunosuppression, Body Mass Index (BMI), and vaccination protocols, including doses and interval between vaccines, were extracted. In addition, the post vaccination humoral response, antibody titer, and rate of adverse events were collected regarding the outcomes. A consensus regarding the differences between the research selection and data extraction was reached through consultation.

### Risk of bias assessment

The risk of bias in the included studies was assessed using the risk of bias in non-randomized studies of interventions (ROBINS-I) (13). There were seven Bias domains included in this scale, each of which was accessed to be “low,” “moderate,” “serious,” “critical,” and “no information.” The opinions of five reviewers were combined and a consensus was reached on these controversial points.

### Data synthesis and analysis

RevMan 5.4 and Stata software were used to conduct the analysis. This study pooled antibody titers, seropositivity, and adverse events in hemodialysis patients who received COVID-19 vaccines as the outcome indices. According to a previously published formula, some data with only median (IQR) coverage to mean ± SD for further analysis was converted (14). This meta-analysis was performed in REVMAN 5.4 and Stata using a random-effects model. A ≥ 50% value of the I^2^ statistic was considered substantially heterogeneous for the pooled estimate. A sensitivity analysis was conducted to identify potential sources of heterogeneity by excluding studies with a high risk of bias. Subgroup analysis was performed to identify age, immunosuppression, dialysis vintage, the prevalence of diabetes, doses, the timing for detecting, continents and vaccine types to clarify the causes of heterogeneity.

## Results

### Study selection and population characteristics

**Figure 1:**
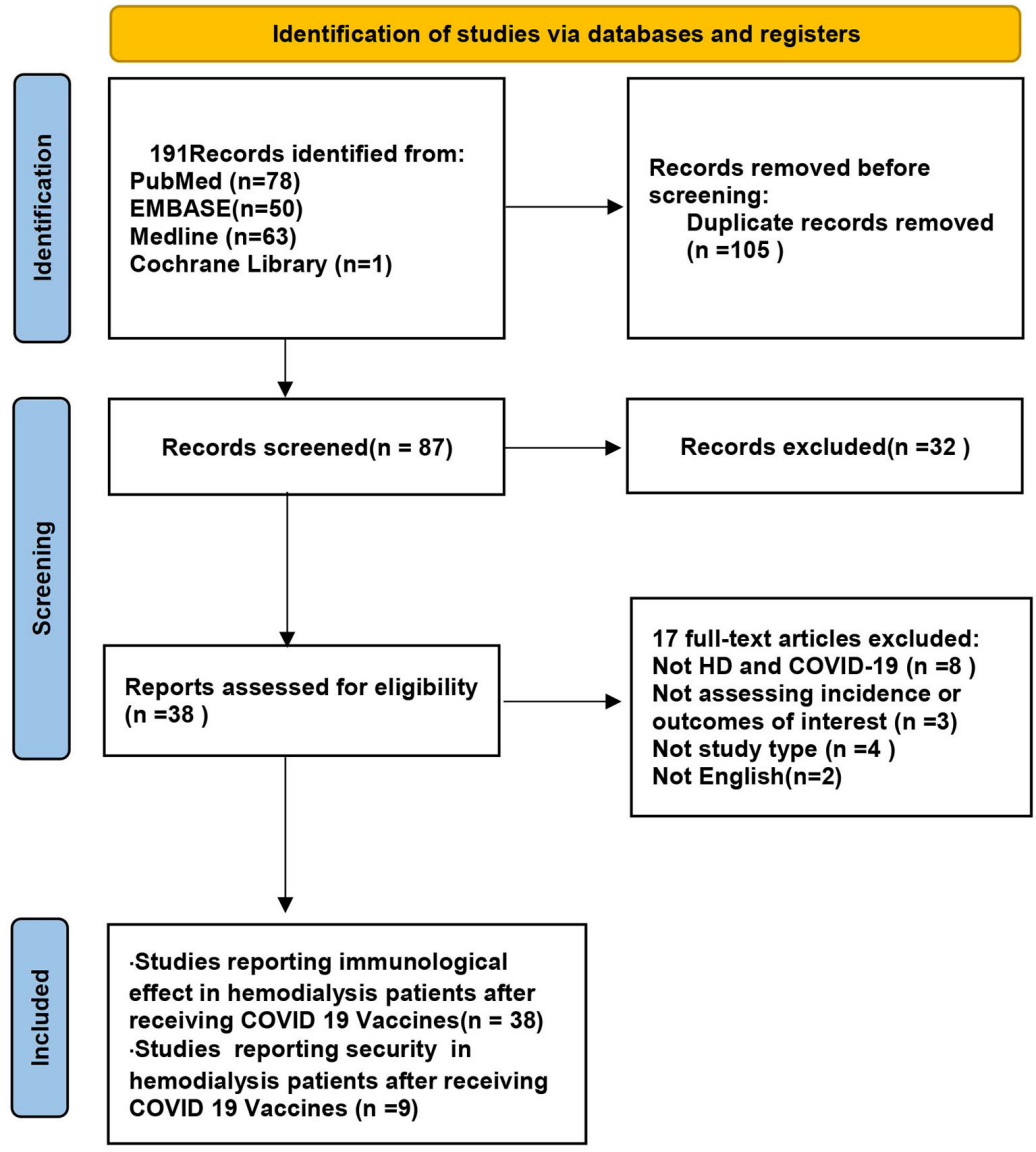
Study select progress of identification of studies via databases and registers.

In this paper, 78, 50, 63, and one potentially eligible article was collected by searching PubMed, EMBASE, MEDLINE, and Cochrane Library, respectively. After reviewing the titles and abstracts, 105 duplicate studies and 32 irrelevant studies were excluded. After reviewing the full text to further determine the study's eligibility, studies whose subjects did not meet the requirements and did not address the results of interest were excluded. Finally, a total of 38 studies investigating immunogenicity, with nine studies investigating vaccine safety, were included (Figure 1). Table 1 summarizes the data extracted from the selected studies (15–52).

**Table 1:** Characteristics of included studies.

**Study**	**Country**	**Population**	**Prior COVID infection**	**Age (Mean±SD)**	**Male (%)**	**BMI (kg/m^2^)**	**Diabetes mellitus N(%)**	**Immuno- suppression N (%)**	**Dialysis vintage (months)**	**Name of vaccine**	**Dose**	**Criteria for positive response**
Agur et al. (15)	Israel	HD/PD	NO	71.57 ± 12.87	33.6	26.69 ± 5.51	70 (57.4%)	NR	39.73 ± 32.59	BNT162b2	2	Anti–spike antibody >50 AU/ml
Anand et al. (16)	USA	HD	Mixed	NR	NR	NR	NR	NR	NR	BNT162b2	2	NR
Attias et al. (17)	France	HD	NO	71 ± 11.5	78.0	NR	33 (58%)	NR	NR	BNT162b2	2	Anti–spike antibody signal-to-cutoff <1
			YES	69 ± 13.5	77.0		7 (54%)					
Bertrand et al. (18)	France	KTRs/HD	NO	71.2 ± 16.4	51.0	NR	NR	NR	NR	BNT162b2	2	Anti–spike antibody >50 AU/ml
Billany et al. (19)	UK	HD	Mixed	62.1 ± 12.2	59.6	NR	43 (45.7%)	10 (10.6%)	NR	BNT162b2 /AZD1222	1	Anti–spike antibody >1 RLU/ml
Broseta et al. (20)	Spain	HD	NO	67.1 ± 16.0	67.9	NR	26 (33.3%)	NR	94.26 ± 127.75	mRNA-1273	2	Anti–spike antibody >50 AU/ml
Chan et al. (21)	USA	HD	NO YES	70 ± 11	93.0	NR	20(49%)	NR	NR	mRNA-1273	2	Anti-N IgG > 1.39, Anti-RBD IgG > 1.0
					100.0		15(75%)					
Clarke et al. (22)	UK	HD	Mixed	NR	NR	NR	NR	NR	NR	BNT162b2 /ChAdOx	2	NR
Cserep et al. (23)	UK	HD	NO	73 ± 11.67*	60.0	NR	31 (37%)	NR	NR	BNT162b2	2	NR
Danthu et al. (24)	France	HD/KTRs/HC	NO	73.5 ± 12.8	59.0	26.8 ± 5	42(53.8%)	NR	NR	BNT162b2	2	antibody>13 AU/ml
Duarte et al. (25)	Portugal	HD	NO	75.1 ± 11.7	59.5	NR	19 (45.2%)	NR	NR	BNT162b2	2	NR
		PD		60.5 ± 10.7								
Ducloux et al. (26)	France	HD/PD	Mixed	NR	NR	NR	NR	NR	NR	BNT162b2	2	Antibody >50 AU/ml
Espi et al. (27)	France	HD/HC	NO	64.9 ± 15.2	65.0	26.5 ± 6.5	37 (35%)	13 (12%)	50.7 ± 60.7	BNT162b2	2	Anti-RBD IgG > 1 AU/ml
Fernando and Govindan (28)	India	HD	NO	NR	NR	NR	NR	NR	NR	AZD1222 /BBV152	2	IgG anti-spike protein >0.8 U/mL
Frantzen et al. (29)	France	HD	NO	71.3 ± 12.7*	70.0	NR	90 (37%)	1(4.1%)	NR	BNT162b2	2	Anti–spike antibody >15 U/ml
Goupil et al. (30)	Canada	HD/HC	NO	70 ± 14	77.0	NR	72 (55%)	22 (16%)	45.6 ± 44.4	BNT162b2	1	NR
			YES	76 ± 12	53.0	NR	7 (37%)	1 (5%)	40.8 ± 38.4			
Grupper et al. (31)	Israel	HD/HC	NO	74 ± 11	75.0	27.2 ± 4	35 (63%)	1 (2%)	38 ± 37	BNT162b2	2	Anti–spike antibody >50 AU/ml
Jahn et al. (32)	Germany	HD/HC	NO	68 ± 8.83*	43.1	NR	NR	NR	62.7 ± 66.0	BNT162b2	2	Antibody ≥13.0AU/mL
Labriola et al. (33)	Belgium	HD/HC	Mixed	80.7 ± 10.0*	44.0	NR	14 (41%)	NR	NR	BNT162b2	2	Anti-SARS-CoV-2 N >1.0 or anti-SARS-CoV-2 RBD >0.8 U/mL
Lacson et al. (34)	USA	HD	NO	68 ± 12	53.0	NR	NR	NR	58.1 ± 54.3	mRNA-1273 / BNT162b2	2	Antibody ≥2.0
Lesny et al. (35)	Germany	HD	NO	69.3 ± 17.4*	55.6	27.9 ± 4.3*	6 (26.1%)	3 (13.0%)	29.7 ± 29.2*	BNT162b2 /AZD1222	1	SARS-CoV-2 spike IgG ≥ 50 AU mL
Longlune et al. (36)	France	HD	NO	64 ± 14	68.8	NR	33(29.5%)	NR	39 ± 40	BNT162b2	2	Spike antibody signal-to-cutoff [S/CO] >1
Mulhern et al. (37)	USA	HD	Mixed	NR	NR	NR	NR	NR	NR	Ad26.COV2.S / mRNA-1273	2	Spike antibody signal-to-cutoff [S/CO] >1
Rincon et al. (38)	Germany	HD/HC	NO	71.3 ± 14.6*	70.0	NR	19(46.3%)	NR	66.0 ± 64.5*	BNT162b2	2	NR
Sattler et al. (39)	Germany	HD/KTRs/HC	NO	67.39 ±11.88	65.4	NR	12 (46.15%)	NR	82.4 ± 60.8	BNT162b2	2	NR
Schrezenmeier et al. (40)	Germany	HD/HC	NO	74 ± 12.4*	69.4	NR	16(44.4%)	NR	64.0 ± 64.9*	BNT162b2	2	Spike antibody signal-to-cutoff [S/CO] >1
Simon et al. (41)	Austria	HD	NO	67 ± 8.67*	55.0	NR	31(38.3%)	9(11.1%)	NR	BNT162b2	2	Antibody> 29 AU/mL
Speer et al. (42)	Germany	HD/HC	NO	78.7 ± 14.8*	60.0	25.3 ± 5.4*	6 (20%)	NR	37.3 ± 45.9*	BNT162b2	2	NR
Speer et al. (43)	Germany	HD/HC	NO	72.75 ± 10.25*	55.0	NR	14 (64%)	NR	84.0 ± 114.1*	BNT162b2	2	NR
Speer et al. (44)	Germany	HD	NO	83 ± 5.4*	63.0	26.0 ± 4.6*	18 (42%)	8 (19%)	50.0 ± 47.6*	BNT162b2	2	A semi-quantitative index of>1
Strengert et al. (45)	Germany	HD/HC	NO	69 ± 18	58.0	NR	22 (27.16%)	10 (12.34%)	NR	BNT162b2	2	NR
Stumpf et al. (46)	Germany	HD/HC	NO	67.6 ± 14	65.1	27.5 ± 5.7	430(34.2%)	63(5%)	68.4 ± 67.2	mRNA-1273 / BNT162b2	2	NR
Torreggiani et al. (47)	France	HD/HC	NO	68.89 ± 14.86	59.0	NR	NR	NR	30.8 ± 34.9	BNT162b2	2	NR
Tylicki et al. (48)	Poland	HD	NO	69.3 ± 10.5	61.5	25.7 ± 5.3*	34 (37.4%)	6 (6.6%)	36.0 ± 34.7	BNT162b2	2	NR
			YES	65.7 ± 12.4	65.7	25.1 ± 4.5*	5 (14.3%)	2 (5.7%)	44.3 ± 52.6	BNT162b2	2	
Weigert et al. (49)	Portugal	HD/HC	NO	64 ± 49.4	67.8	NR	NR	NR	NR	BNT162b2	2	NR
Yanay et al. (50)	Israel	HD /PD/HC	NO	69.7 ± 12	63.0	NR	NR	NR	40.8 ± 34.0	BNT162b2	2	Anti–spike antibody >15 AU/ml
Yau et al. (51)	Canada	HD/HC	NO	73.7 ± 13.6	61.0	NR	45 (59%)	4 (5%)	35.2 ± 27.2	BNT162b2	2	NR
Zitt et al. (52)	Austria	HD	NO	67.6 ± 14.8	68.0	NR	NR	NR	NR	BNT162b2	2	NR

* Data are converted from the median (IQR) according to the formula in the article. Estimating the sample mean and standard deviation from the sample size, median, range, and/or interquartile range.

Among the 38 included studies, 20 were prospective observational studies, four were retrospective studies, and one was a cross-sectional study (13 studies did not state the research types). Thirty-seven of the included studies reported the seropositivity rate in hemodialysis patients 1–8 weeks after receiving COVID-19 vaccine. On average, 17 of 38 studies compared the immunogenicity of dialysis patients with that of healthy volunteers. Seven studies examined the immunogenicity of dialysis patients with or without prior COVID-19. Six vaccine types (BNT162b2, AZD1222, mRNA-1,276, ChAdOx, BBV152, and Ad26.COV2. S) were studied to determine their immunological effects in HD patients.

The security of COVID-19 vaccines was evaluated by including indices of new infections, survival rates, and adverse events. Two studies reported the rate of new infections, three reported survival rates, and nine reported a variety of local and systemic adverse events.

### Risk of bias assessment

Thirty-eight non-randomized studies were assessed by the Risk of Bias in Non-randomized Studies of Interventions (ROBINS-I) (13). Among the 38 studies, 22 were rated as having a low risk of bias, 10 as moderate risk, and four as severe risk. Two other studies were classified as “no information” due to insufficient data (Supplementary Table S1).

Most questions in the included studies were precise and relevant to the goals of this study. Moreover, most studies collected data according to a previously developed protocol. In some studies, the reasons for exclusion were not specified. Several studies did not indicate how objective endpoints were evaluated and how the study size was calculated.

### Immunogenicity of HD patients after receiving COVID-19 vaccine

**Figure 2:**
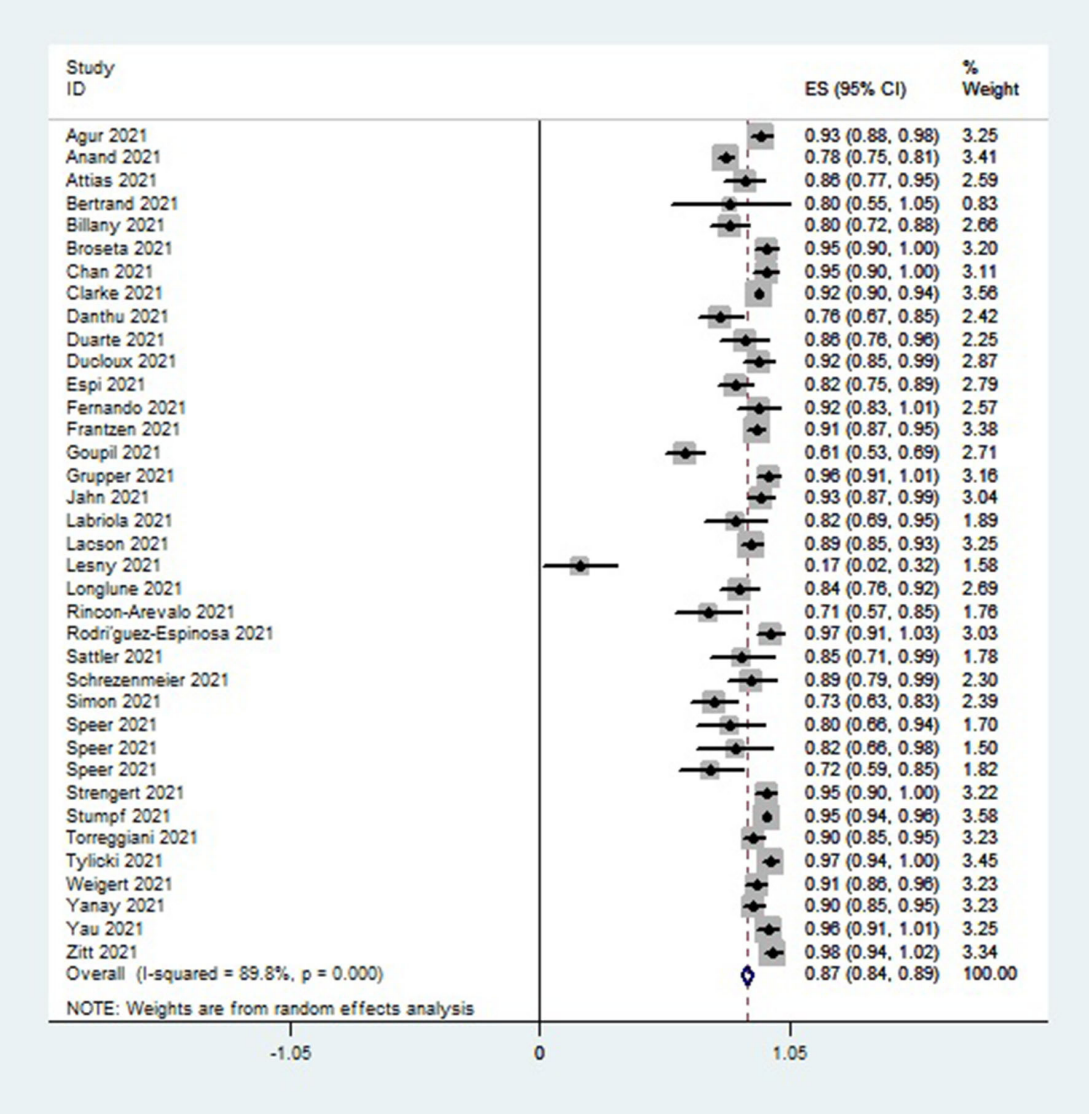
Forest plot of the immune response rate of HD patients who received COVID 19 vaccines, as obtained using Stata.

A single-group meta-analysis of seropositivity rates of hemodialysis patients 2–8 weeks after vaccination revealed overall immunogenicity of 87% (95 CI, 84–89%) with high heterogeneity of I^2^ = 89.8%, as illustrated in Figure 2.

**Figure 3:**
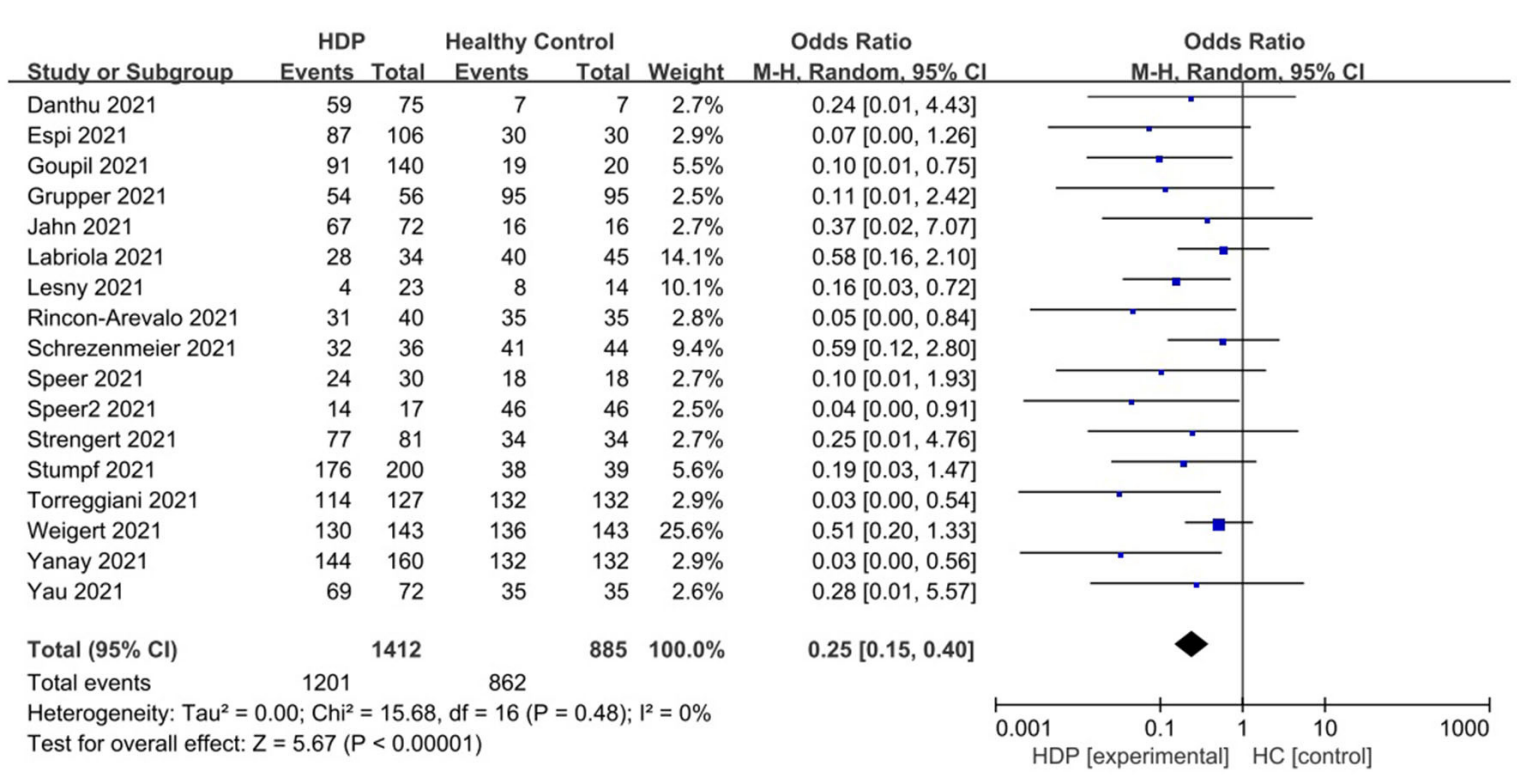
Forest plot of the positive immunity rate of HD patient vs. healthy control groups after receiving COVID 19 vaccines.

As shown in Figure 3, the vaccine response rate in hemodialysis patients (HDPs) was significantly higher than that in healthy control groups (HCs). In HDPs, seropositivity was achieved in approximately 85.1% (1,201 out of 1,402) cases, whereas in HCs, it was achieved in 97.4% (862 out of 885) cases.

**Figure 4:**
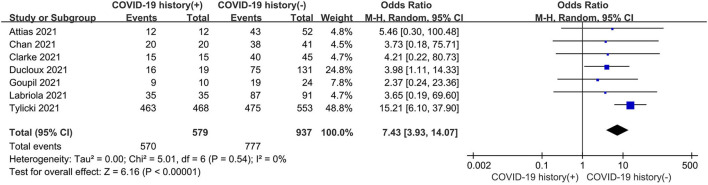
Forest plot of the immune response rate of HD patients with or without previous COVID-19 infection.

**Figure 5:**
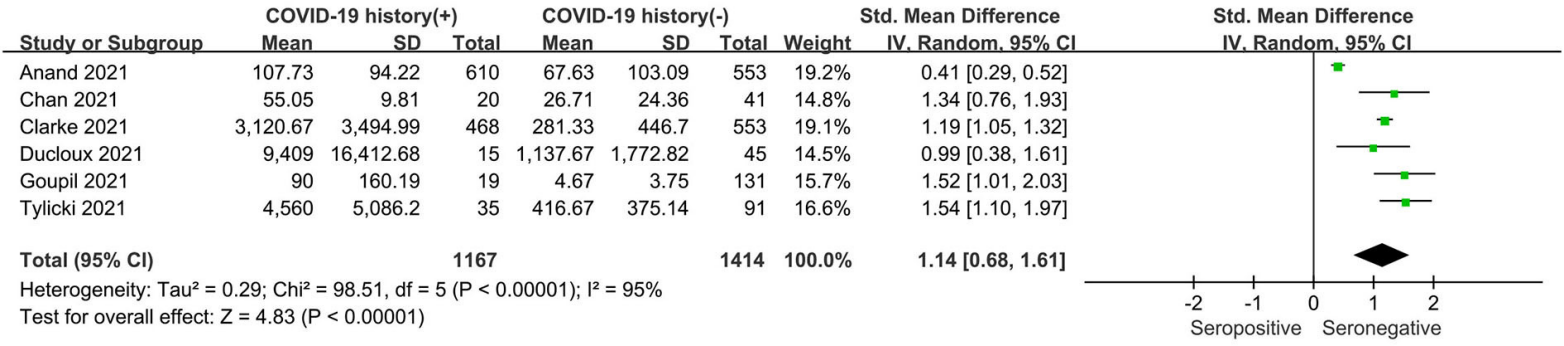
Forest plot of the antibody titer of HD patients with or without previous COVID-19 infection.

As shown in Figure 4, the seropositive conversion rate in patients without prior infection was lower than in patients with prior infection. It was 82.9% (777 of 937) in infection-naive patients and 98.4% (570 of 579) in patients with previous infections. Furthermore, antibody titers were compared among dialysis patients with and without prior COVID-19 infection, and the SMD was 1.14 (95% CI, 0.68–1.61), indicating that patients with prior infection are more likely to develop antibodies (Figure 5).

### Sensitivity analysis

A sensitivity analysis was performed on the included studies by excluding individual studies. After removing each study from the analysis, the seropositivity rate showed no significant difference in the degree of heterogeneity. However, in terms of antibody titer, sensitivity analysis showed that heterogeneity was significantly reduced when one of the studies, Anand et al. (13), was removed. There was a change in the standard mean difference from 1.06 (95% CI, 0.56–1.57) to 1.24 (95% CI, 1.11–1.38), with a reduction in heterogeneity from 95 to 5% (Supplementary Figure S1). This may be because the study was performed in the early phase of vaccine rollout, with the elderly population and patients with complications being prioritized.

### Subgroup analysis

**Figure 6:**
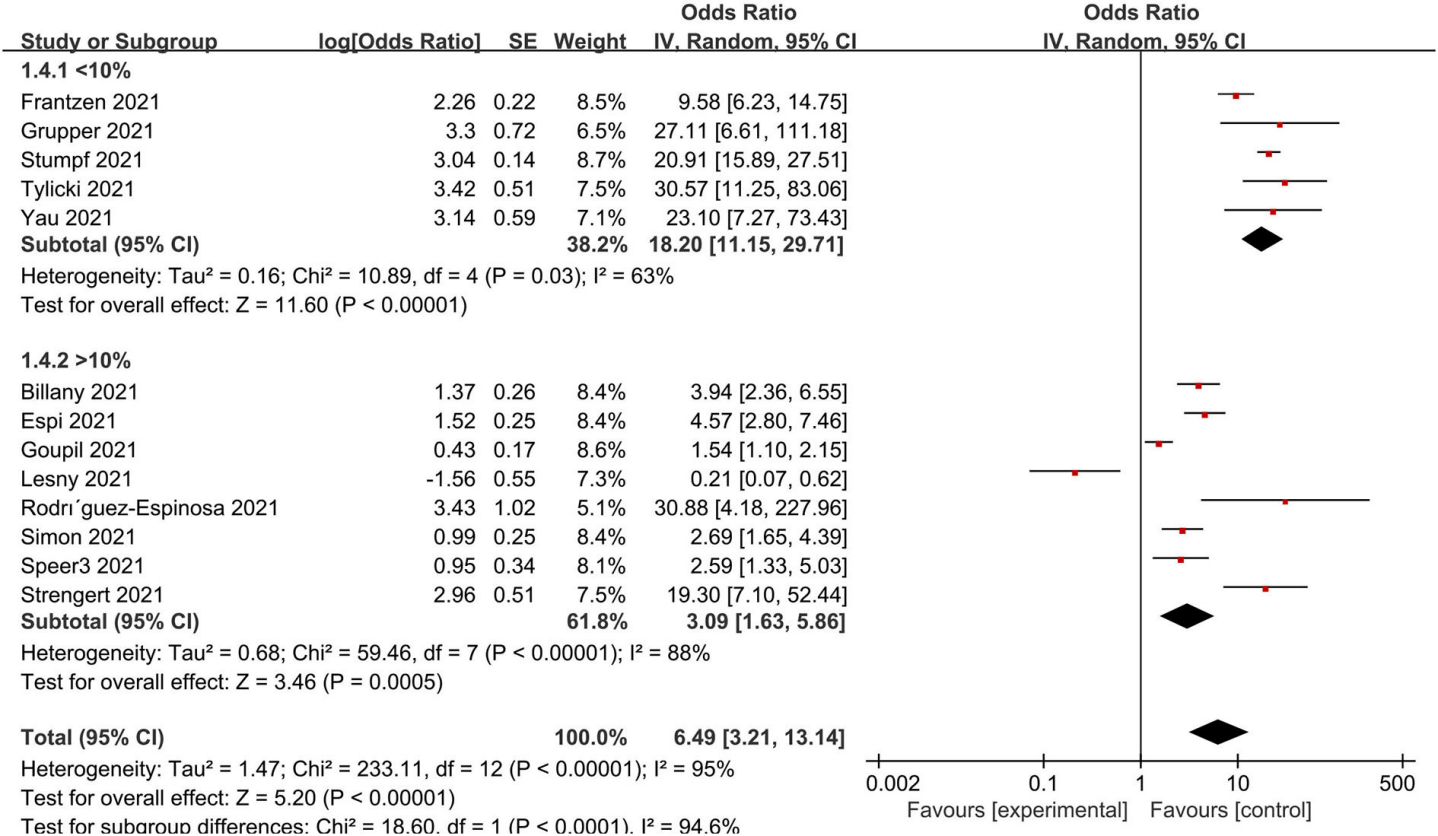
Subgroup analysis of immunosuppressant utilization rate.

A subgroup analysis was performed for age, immunosuppression, dialysis vintage and, the prevalence of diabetes, doses, the timing for detecting, continents and vaccine types to clarify the causes of heterogeneity to identify the possible sources of heterogeneity. Accordingly, low immunosuppression was defined as a rate <10% and high immunosuppression as a rate of 10%. As a result, the population with low immunosuppressive drug utilization rates is more likely to develop immunity to the virus (Figure 6). When studies were grouped according to doses, serological positivity was significantly higher in patients who received two doses of the vaccine than in those who did not complete two doses (Supplementary Figure S2). In addition, the forest plot of the age subgroups (Supplementary Figure S3) revealed no difference between the young (<70 years of age) and old (>70 years of age) groups. A further division was made between dialysis duration <36 months and dialysis duration ≥36 months. No statistically significant difference in dialysis vintage was observed (Supplementary Figure S4). Additionally, there was no significant correlation between the prevalence of diabetes mellitus and the serum positivity rate of the population (Supplementary Figure S5). Further studies showed that factors such as the type of vaccine, the time of testing after vaccination, and country and region were not the sources of heterogeneity in results (Supplementary Figures S6–S8).

### Safety and adverse events

Nine studies examined the safety indices and adverse events following vaccination (20, 25, 33, 38, 41, 42, 47–49). The survival rate of dialysis patients receiving vaccination was high, and there were few deaths due to COVID-19. An extremely low rate of newly acquired infections was observed.

The following local adverse events were observed: pain at the injection site, redness, bruising, and swelling (Table 2). Pain at the injection site was the most common adverse event, accounting for 25% (95 CI, 11–40%), while other local reactions were sporadic (Supplementary Figure S9). Most adverse reactions were mild-to-moderate.

**Table 2:** Local adverse events in dialysis patients after vaccine administration.

**Study**	**Population**	**Sample size**	**New infection *N* (%)**	**Survival rate (%)**	**Pain at the injection site (%)**	**Redness (%)**	**Local bruising (%)**	**Swelling (%)**
Cserep et al. (23)	HD	83	0	100%	NR	NR	4 (4.8%)	2 (2.4%)
Fernando and Govindan (28)	HD	42	2 (4.76%)	97.6%	NR	NR	NR	NR
Longlune et al. (36)	HD	82	0	NR	15 (18.3%)	NR	NR	NR
Simon et al. (41)	HD	81	NR	NR	NR	NR	NR	NR
Speer et al. (44)	HD	43	NR	NR	4 (9.3%)	NR	NR	NR
Strengert et al. (45)	HD	81	NR	100%	NR	NR	NR	NR
Yanay et al. (50)	HD/PD	160	6 (3.75%)	NR	NR	NR	NR	NR
Yau et al. (51)	HD/HC	70	NR	NR	30 (42.9%)	6 (8.6%)	NR	6 (8.6%)
Zitt et al. (52)	HD	48	NR	NR	16 (33.4%)	NR	NR	NR

System adverse events that occurred less frequently were summarized (Table 3), which included fatigue, headaches, fevers, chills, nausea, diarrhea, muscle aches, and joint pain. The most common system adverse reaction was fatigue, accounting for 11% of all adverse reactions (95% CI, 6–18%) (Supplementary Figure S10).

**Table 3:** System adverse events in dialysis patients after vaccine administration.

**Study**	**Population**	**Sample size**	**Fatigue (%)**	**Headache (%)**	**Fever and chills (%)**	**Nausea or vomiting (%)**	**Diarrhea (%)**	**Muscle ache (%)**	**Joint pain (%)**
Cserep et al. (23)	HD	83	9 (10.8%)	7 (8.4%)	3 (3.6%)	NR	1 (1.2%)	NR	NR
Fernando and Govindan (28)	HD	42	NR	NR	NR	NR	NR	NR	NR
Longlune et al. (36)	HD	82	15 (18.3%)	NR	7 (8.5%)	NR	NR	NR	NR
Simon et al. (41)	HD	81	NR	NR	NR	NR	NR	NR	NR
Speer et al. (44)	HD	43	2 (4.6%)	2 (4.6%)	NR	NR	NR	1 (2.3%)	NR
Strengert et al. (45)	HD	81	NR	NR	NR	NR	NR	NR	NR
Yanay et al. (50)	HD/PD	160	NR	NR	NR	NR	NR	NR	NR
Yau et al. (51)	HD/HC	70	15 (21.4%)	NR	9 (12.9%)	6 (8.6%)	3 (4.2%)	NR	6 (8.5%)
Zitt et al. (52)	HD	48	2 (4.2%)	2 (4.2%)	2 (4.2%)	0 (0%)	1 (2.1%)	1 (2.1%)	2 (4.2%)

## Discussion

This review summarized recent studies on the efficacy and safety of COVID-19 vaccine in dialysis patients, so as to provide scientific guidance for clinical vaccination and application. It highlights that the vaccines elicited an adequate immune response in most patients, indicating it to be a sturdy shield to protect patients from the virus, despite the lower immunogenicity rates compared to healthy populations, which is consistent with many previous studies (15, 18, 20).

The low immunogenicity rate as well as inadequate innate and adaptive immune responses in dialysis patients is caused by a combination of reasons (53). In terms of pathogenesis, the progression of CKD is closely related to the dysfunction of autoimmune system, as the deposition of immune complexes will cause damage to the basement membrane. For current therapeutic interventions, the high rate of prolonged immunosuppressant use can hinder adaptive immune responses and contribute to COVID-19 severity (54). A retrospective study concluded that COVID-19 disease is more severe in patients taking prior immunosuppressive medications as the data showed that patients with COVID-19 having prior immunosuppressive therapy had significantly greater mortality, longer lengths of hospitalization, and longer ICU stays (55). Based on our subgroup analysis, a negative correlation between immunosuppression and immune response was found. Accordingly, the higher the rate of herd immunosuppression, the lower the immune response. This may explain why the response rate of dialysis patients is lower than that of healthy individuals. Additionally, the dialysis procedures make it inevitable to lose some immune protein factors in the process of dialysis. Furthermore, the characteristics of multiple complications in dialysis patients will also cause the low immunogenicity rate.

The characteristics of the dialysis population may contribute to immunogenicity acquisition as well. Several factors have been reported to be associated with immune responses, including age (30, 32), body mass index (BMI) (15), previous immunosuppression (24, 36), dialysis vintage, and diabetes prevalence (19). To validate the conclusions of previous studies, we performed subgroup analyses of age, dialysis vintage and diabetes prevalence, but the data obtained did not support us to draw similar conclusions. Additionally, data on BMI was collected, the mean of which was quite similar, fluctuating around 27 kg/m^2^; thus, subgroup analyses of this type of data were not performed. From our perspective, large sample studies and sufficient data are needed to support the effects of these factors.

Vaccination schedules also affect the rate of immunogenicity in patients. Taking vaccine dose, type and testing time into consideration, the data revealed that the immune response rate was correlated with the dose rather than vaccine type and timing of detecting, which was consistent with previous reports (26, 28, 35).

In the current study, the immune response rate was higher in patients with a prior infection than in patients who had not been infected. With respect to the first immune response, the second immune response is characterized by a shorter incubation period, increased antibody levels, and a more extended maintenance period, which can explain the difference between the immune responses of patients with and without infection histories (56).

Vaccine safety studies are few, and statistics are lacking. Based on available data, the survival rate of COVID-19 vaccinated patients was close to 100%, although some patients still developed new infections (<5%) (23, 28, 35, 50). The most commonly reported adverse event is local pain at the injection site. Previous studies have also reported redness, local bruising, and swelling (23, 36, 44, 51, 52). System adverse events included fatigue, headache, fever and chills, nausea or vomiting, diarrhea, muscle aches, and joint pain, among which fatigue was the most common (23, 36, 44, 51, 52). Based on the data analysis, occasional adverse events do not threaten dialysis patients' safety.

The review has certain limitations. The literature included in this systematic review mainly included the data of the first two doses of COVID-19 vaccine. With the continuous development of COVID-19 vaccine, booster doses have been carried out and obtained in many countries. The discussion of the booster doses was lacking in this study. With the development of multi-center, large-sample studies worldwide, more comprehensive summaries are expected.

Overall, this system review focused on the effect and safety of COVID-19 vaccines. From the perspective of this study, dialysis patients in stable health conditions are encouraged to receive vaccines. For dialysis patients, COVID-19 vaccination is more beneficial than risky. Although malignancies occasionally occur, these adverse effects have little impact on health, and the antibodies produced by the vaccines can effectively protect the body against the virus. Of note, in order to obtain sufficient immune protection for these special individuals, alternative strategies need to be innovated and developed.

## Conclusion

Overall, the systematic meta-analysis confirmed the positive effects of COVID-19 vaccine in dialysis patients. Although it is less efficient than in healthy people, it can protect the patient's body from the virus to some degree. Furthermore, it can be concluded that the vaccine is safe for dialysis patients due to the low incidence of adverse events and absence of life-threatening incidents.

Considering our viewpoint, it could be a reasonable option for dialysis patients in stable condition to receive COVID-19 vaccine, which can prevent the transmission of the virus. However, strict post-vaccination observation is necessary to ensure the safety of patients and to avoid the occurrence of serious malignant events. Therefore, an optimal treatment plan should be discussed comprehensively based on the patient's specific characteristics for patients who have used immunosuppressants for an extended period.

## Data availability statement

The original contributions presented in the study are included in the article/Supplementary material, further inquiries can be directed to the corresponding authors.

## Author contributions

RP, YM, and SX contributed equally to the intellectual content during manuscript drafting and took responsibility for the accuracy and integrity of the materials, performed the statistical analysis, and drafted of the manuscript. RP and ZY designed the study protocol. RP and YM performed the electronic database search. YM, SX, HW, and ZD screened the citations retrieved from electronic searches. ZY, JJ, HQ, RP, YM, SX, ZD, and HW weighed on the bias of the studies, consulted on discrepancies during screening, and data extraction. RP, YM, SX, HW, and ZD performed data extraction, synthesis, and interpretation. ZY, JJ, and HQ supervision and obtained funding. All authors contributed to the article and approved the submitted version.

## Funding

This research was supported by the Huadong Medicine Joint Funds of the Zhejiang Provincial Natural Science Foundation of China (Grant No. LHDMZ22H050001), the Construction of Key Projects by the Zhejiang Provincial Ministry (Project No. WKJ-ZJ-2017), the Zhejiang Province Chinese Medicine Modernization Program (Project No. 2020ZX001), Key Project of Scientific Research Foundation of Chinese Medicine (2022ZZ002), Key Project of Zhejiang Science and Technology Department (202203118), Key Project of Basic Scientific Research Operating Funds of Hangzhou Medical College (KYZD202002), and Young Talent Project Funding from the Health Commission of Zhejiang Province (No. 2019RC008).

## Conflict of interest

The authors declare that the research was conducted in the absence of any commercial or financial relationships that could be construed as a potential conflict of interest.

## Publisher's note

All claims expressed in this article are solely those of the authors and do not necessarily represent those of their affiliated organizations, or those of the publisher, the editors and the reviewers. Any product that may be evaluated in this article, or claim that may be made by its manufacturer, is not guaranteed or endorsed by the publisher.
