# Supplementary material for: Immunogenicity and safety of SARS-CoV-2 vaccine in hemodialysis patients: A systematic review and meta-analysis
Source: Front Public Health. 2022 Sep 23;10:951096. doi: 10.3389/fpubh.2022.951096 (PMC9539993; doi:10.3389/fpubh.2022.951096)

Supplementary Material

## Supplementary Item S1: Electronic database search strategies

**1.1 PubMed:**

#1 (((((((((Renal Dialysis [MeSH Terms]) OR (Dialyses, Renal)) OR (Renal Dialyses)) OR (Dialysis, Renal)) OR (Hemodialysis)) OR (Hemodialyses)) OR (Dialysis, Extracorporeal))

OR (Dialyses, Extracorporeal)) OR (Extracorporeal Dialyses)) OR (Extracorporeal Dialysis)

#2 (((((((((((((((((((((((((((((((((((((((((((((((((((((COVID-19 Vaccines[MeSH Terms]) OR COVID 19 Vaccines])) OR (Vaccines,COVID-19)) OR (COVID-19 Virus Vaccines)) OR (COVID 19 Virus Vaccines)) OR (Vaccines, COVID-19 Virus)) OR (Virus Vaccines, COVID-19)) OR (COVID-19 Virus Vaccine)) OR (COVID 19 Virus Vaccine)) OR ( Vaccine, COVID-19 Virus)) OR (Virus Vaccine, COVID-19)) OR (COVID19 Virus Vaccines)) OR (Vaccines, COVID19 Virus)) OR ( Virus Vaccines, COVID19)) OR (COVID19 Virus Vaccine)) OR ( Vaccine, COVID19 Virus)) OR (Virus Vaccine, COVID19)) OR (COVID19 Vaccines)) OR (Vaccines, COVID19)) OR ( COVID19 Vaccine)) OR ( Vaccine, COVID19)) OR (SARS-CoV-2 Vaccines)) OR ( SARS CoV 2 Vaccines)) OR ( Vaccines, SARS-CoV-2)) OR ( SARS-CoV-2 Vaccine)) OR ( SARS CoV 2 Vaccine)) OR (Vaccine, SARS-CoV-2)) OR (SARS2 Vaccines)) OR (Vaccines, SARS2)) OR (SARS2 Vaccine)) OR ( Vaccine, SARS2)) OR ( Coronavirus Disease 2019 Vaccines)) OR ( Coronavirus Disease 2019 Vaccine)) OR ( Coronavirus Disease 2019 Virus Vaccine)) OR (Coronavirus Disease 2019 Virus Vaccines)) OR (Coronavirus Disease-19 Vaccines)) OR ( Coronavirus Disease 19 Vaccines)) OR ( Vaccines, Coronavirus Disease-19)) OR ( Coronavirus Disease-19 Vaccine)) OR ( Coronavirus Disease 19 Vaccine)) OR ( Vaccine, Coronavirus Disease-19)) OR ( COVID 19 Vaccine)) OR (Vaccine, COVID 19)) OR ( 2019-nCoV Vaccine)) OR ( 2019 nCoV Vaccine)) OR ( Vaccine, 2019-nCoV)) OR ( 2019 Novel Coronavirus Vaccines)) OR (2019 Novel Coronavirus Vaccine)) OR (2019-nCoV Vaccines)) OR ( 2019 nCoV Vaccines)) OR (Vaccines, 2019-nCoV)) OR ( COVID-19 Vaccine)) OR ( Vaccine, COVID-19)) OR (SARS Coronavirus 2 Vaccines)

#3 #1 AND #2

Results:78

**1.2 EMBASE:**

#1 'hemodialysis'/exp

#2 ‘Dialyses, Renal’ OR ‘Renal Dialyses’ OR ‘Dialysis, Renal’ OR ‘Hemodialysis’ OR ‘ Hemodialyses’ OR ‘Dialysis, Extracorporeal’ OR ‘Dialyses, Extracorporeal’ OR ‘Extracorporeal Dialyses’ OR ‘Extracorporeal Dialysis’

#3 #1 OR #2

#4 'SARS-CoV-2 vaccine' OR ' /exp

#5 'COVID 19 Vaccines' OR 'Vaccines, COVID-19' OR 'COVID-19 Virus Vaccines' OR 'COVID 19 Virus Vaccines' OR 'Vaccines, COVID-19 Virus' OR 'Virus Vaccines, COVID-19' OR 'COVID-19 Virus Vaccine' OR 'COVID 19 Virus Vaccine' OR 'Vaccine, COVID-19 Virus' OR 'Virus Vaccine, COVID-19' OR 'COVID19 Virus Vaccines' OR 'Vaccines, COVID19 Virus' OR 'Virus Vaccines, COVID19' OR 'COVID19 Virus Vaccine' OR 'Vaccine, COVID19 Virus' OR 'Virus Vaccine, COVID19' OR 'COVID19 Vaccines' OR 'Vaccines, COVID19' OR 'COVID19 Vaccine' OR 'Vaccine, COVID19' OR 'SARS-CoV-2 Vaccines' OR 'SARS CoV 2 Vaccines' OR 'Vaccines, SARS-CoV-2' OR 'SARS-CoV-2 Vaccine' OR 'SARS CoV 2 Vaccine' OR 'Vaccine, SARS-CoV-2' OR 'SARS2 Vaccines' OR 'Vaccines, SARS2' OR 'SARS2 Vaccine' OR 'Vaccine, SARS2' OR 'Coronavirus Disease 2019 Vaccines' OR 'Coronavirus Disease 2019 Vaccine' OR 'Coronavirus Disease 2019 Virus Vaccine' OR 'Coronavirus Disease 2019 Virus Vaccines' OR 'Coronavirus Disease-19 Vaccines' OR 'Coronavirus Disease 19 Vaccines' OR 'Vaccines, Coronavirus Disease-19' OR 'Coronavirus Disease-19 Vaccine' OR 'Coronavirus Disease 19 Vaccine' OR 'Vaccine, Coronavirus Disease-19' OR 'COVID 19 Vaccine' OR 'Vaccine, COVID 19' OR '2019-nCoV Vaccine' OR '2019 nCoV Vaccine' OR 'Vaccine, 2019-nCoV' OR '2019 Novel Coronavirus Vaccines' OR '2019 Novel Coronavirus Vaccine' OR '2019-nCoV Vaccines' OR '2019 nCoV Vaccines' OR 'Vaccines, 2019-nCoV' OR 'COVID-19 Vaccine' OR 'Vaccine, COVID-19' OR 'SARS Coronavirus 2 Vaccines'

#6 #4 OR #5

#7 #3 AND #6

Results: 50

**1.3 MEDLINE:**

#1 ((((((((((((((((((((((((((((((((((((((((((((((((((((TS=(COVID 19 Vaccines)) OR TS=(Vaccines, COVID-19)) OR TS=(COVID-19 Virus Vaccines)) OR TS=(COVID 19 Virus Vaccines)) OR TS=(Vaccines, COVID-19 Virus)) OR TS=(Virus Vaccines, COVID-19)) OR TS=(COVID-19 Virus Vaccine)) OR TS=(COVID 19 Virus Vaccine)) OR TS=(Vaccine, COVID-19 Virus)) OR TS=(Virus Vaccine, COVID-19)) OR TS=(COVID19 Virus Vaccines)) OR TS=(Vaccines, COVID19 Virus)) OR TS=(Virus Vaccines, COVID19)) OR TS=(COVID19 Virus Vaccine)) OR TS=(Vaccine, COVID19 Virus)) OR TS=(Virus Vaccine, COVID19)) OR TS=(COVID19 Vaccines)) OR TS=(Vaccines, COVID19)) OR TS=(COVID19 Vaccine)) OR TS=(Vaccine, COVID19)) OR TS=(SARS-CoV-2 Vaccines)) OR TS=(SARS CoV 2 Vaccines)) OR TS=(Vaccines, SARS-CoV-2)) OR TS=(SARS-CoV-2 Vaccine)) OR TS=(SARS CoV 2 Vaccine)) OR TS=(Vaccine, SARS-CoV-2)) OR TS=(SARS2 Vaccines)) OR TS=(Vaccines, SARS2)) OR TS=(SARS2 Vaccine)) OR TS=(Vaccine, SARS2)) OR TS=(Coronavirus Disease 2019 Vaccines)) OR TS=(Coronavirus Disease 2019 Vaccine)) OR TS=(Coronavirus Disease 2019 Virus Vaccine)) OR TS=(Coronavirus Disease 2019 Virus Vaccines)) OR TS=(Coronavirus Disease-19 Vaccines)) OR TS=(Coronavirus Disease 19 Vaccines)) OR TS=(Vaccines, Coronavirus Disease-19)) OR TS=(Coronavirus Disease-19 Vaccine)) OR TS=(Coronavirus Disease 19 Vaccine)) OR TS=(Vaccine, Coronavirus Disease-19)) OR TS=(COVID 19 Vaccine)) OR TS=(Vaccine, COVID 19)) OR TS=(2019-nCoV Vaccine)) OR TS=(2019 nCoV Vaccine)) OR TS=(Vaccine, 2019-nCoV)) OR TS=(2019 Novel Coronavirus Vaccines)) OR TS=(2019 Novel Coronavirus Vaccine)) OR TS=(2019-nCoV Vaccines)) OR TS=(2019 nCoV Vaccines)) OR TS=(Vaccines, 2019-nCoV)) OR TS=(COVID-19 Vaccine)) OR TS=(Vaccine, COVID-19)) OR TS=( SARS Coronavirus 2 Vaccines)

#2 MH=Dialysis

#3 TS=Renal Dialyses

#4 TS=Dialysis, Renal

#5 TS= Dialyses, Renal

#6 TS= Renal Dialyses

#7 TS=Dialysis, Renal

#8 TS=Hemodialysis

#9 TS=Hemodialyses

#10 TS=Dialysis, Extracorporeal

#11 TS=Dialyses, Extracorporeal

#12 TS=Extracorporeal Dialyses

#13 TS=Extracorporeal Dialysis

#14 #2 OR #3 OR #4 OR #5 OR #6 OR #7 OR #8 OR #9 OR #10 OR #11 OR #12 OR #13

#15 #14 AND #1

Result=63

**1.4 Cochrane Library:**

#1 MeSH descriptor: [Renal Dialyses] explode all trees

#2 MeSH descriptor: [COVID 19 Vaccines] explode all trees

#3 (Dialys*): ti,ab,kw

#4 (Hemodialys*):ti,ab,kw

#5 #3 OR #4 OR #1

#6 (2019 Novel Coronavirus*): ti,ab,kw

#7 (SARS CoV 2 Virus*): ti,ab,kw

#8 (SARS-CoV-2 Virus*): ti,ab,kw

#9 (COVID-19 Virus*): ti,ab,kw

#10 (Severe Acute Respiratory Syndrome Coronavirus 2):ti,ab,kw

#11 #6 OR #7 OR #8 OR #9 OR #10

#12 MeSH descriptor: [Vaccines] explode all trees

#13 (vaccine): ti,ab,kw

#14 #12 OR #13

#15 #11 AND #14

#16 #2 OR #15

#17 #5 AND #16

Results:1

## Table S1: Risk of bias assessment of included study by ROBINS-I (Risk of Bias in Non-randomized Studies of Interventions)

| Study | Bias due to  confounding | Bias in selection of  participants into the study | Bias in classification of  interventions | Bias due to deviations  from intended  interventions | Bias due to missing  data | Bias in measurement of  outcomes | Bias in selection of the  reported result | Overall risk of bias assessment |
| --- | --- | --- | --- | --- | --- | --- | --- | --- |
| Agur 2021 | Moderate | Low | Low | Low | Low | Moderate | Low | Low |
| Anand 2021 | Serious | Low | Low | Moderate | Serious | Low | Low | Serious |
| Attias 2021 | Moderate | Low | Low | Low | Low | Low | Low | Low |
| Bertrand 2021 | Moderate | Low | Low | Low | Moderate | Low | Low | Moderate |
| Billany 2021 | Serious | Low | Low | Low | Low | Low | Low | Moderate |
| Broseta 2021 | Low | Moderate | No information | Low | Low | Low | Low | Low |
| Chan 2021 | Serious | Low | Low | Low | Low | Low | Low | Low |
| Clarke 2021 | Serious | Low | Moderate | Low | Serious | Low | Low | Serious |
| Cserep 2021 | Moderate | Moderate | Moderate | Moderate | Moderate | Low | Low | Moderate |
| Danthu 2021 | Moderate | Moderate | Low | Serious | Low | Low | Low | Moderate |
| Duarte 2021 | Serious | Low | Low | Low | Low | Low | Low | Low |
| Ducloux 2021 | No information | Low | No information | Low | Moderate | Low | ;ow | Moderate |
| Espi 2021 | Low | Low | Low | Low | Low | Low | Low | Low |
| Fernando 2021 | Serious | Low | No information | Low | Moderate | Low | Low | Low |
| Frantzen 2021 | No information | No information | No information | Moderate | Moderate | Moderate | Low | Serious |
| Goupil 2021 | Moderate | Low | Low | Low | Low | Low | Low | Low |
| Grupper 2021 | Low | Moderate | Low | Low | Low | Low | Low | Low |
| Jahn 2021 | Low | Low | Low | Low | Low | Low | Low | Low |
| Labriola 2021 | Moderate | Low | Low | Low | Low | Low | Low | Low |
| Lacson 2021 | No information | Moderate | Low | Low | No information | Moderate | Low | Moderate |
| Lesny 2021 | Moderate | Low | Low | Serious | Serious | Low | Low | Serious |
| Longlune 2021 | Low | Low | Low | Low | Low | Low | Low | Low |
| Mulhern 2021 | No information | No information | Low | Moderate | Low | Low | Low | Low |
| Rincon 2021 | Moderate | Moderate | Low | Low | Low | No information | Low | Moderate |
| Sattler 2021 | Low | Moderate | Low | Low | Low | Low | Low | Low |
| Schrezenmeier 2021 | Low | Low | Low | Low | Low | Low | Low | Low |
| Simon 2021 | No information | Low | Low | Low | Low | Low | Low | Low |
| Speer1 2021 | No information | Low | No information | Low | Low | No information | Low | No information |
| Speer2 2021 | Low | Low | Low | Low | Low | Low | Low | Low |
| Speer3 2021 | Low | Low | Low | Moderate | Serious | Low | Low | Moderate |
| Strengert 2021 | No information | Low | Low | Moderate | Moderate | Low | Low | Moderate |
| Stumpf 2021 | Low | Low | Low | Low | Low | Low | Low | Low |
| Torreggiani2021 | No information | Low | Low | Moderate | Low | Low | Low | Low |
| Tylicki 2021 | No information | Low | Low | Moderate | Low | Low | Moderate | Moderate |
| Weigert 2021 | Low | Low | Low | Low | Low | Low | Low | Low |
| Yanay 2021 | No information | Low | Low | No information | No information | Low | No information | No information |
| Yau 2021 | Low | Low | Low | Low | Low | Low | Low | Low |
| Zitt 2021 | No information | Low | Low | Low | Low | Low | Low | Low |

## Figure S1: Sensitivity analysis of antibody titer of HD patients who received COVID 19 Vaccines


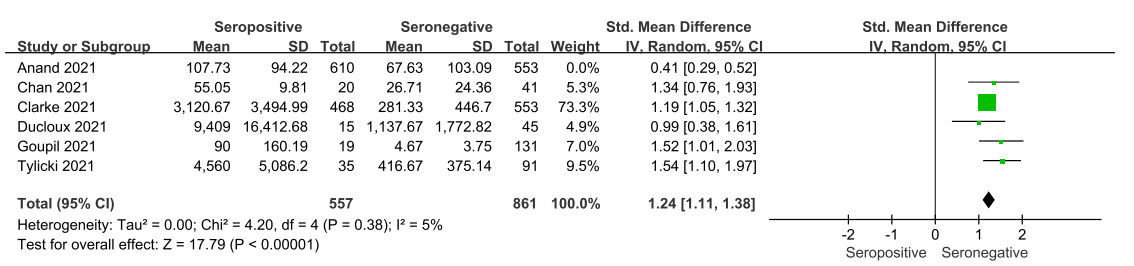


## Figure S2：Subgroup analysis of vaccine doses

##
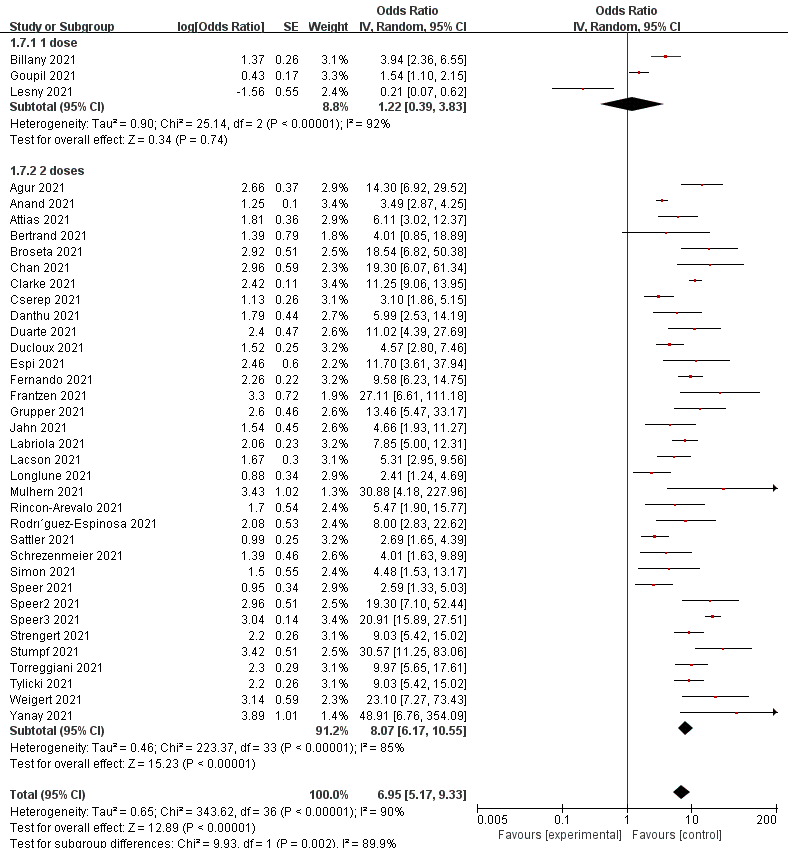


## Figure S3: Subgroup analysis by age


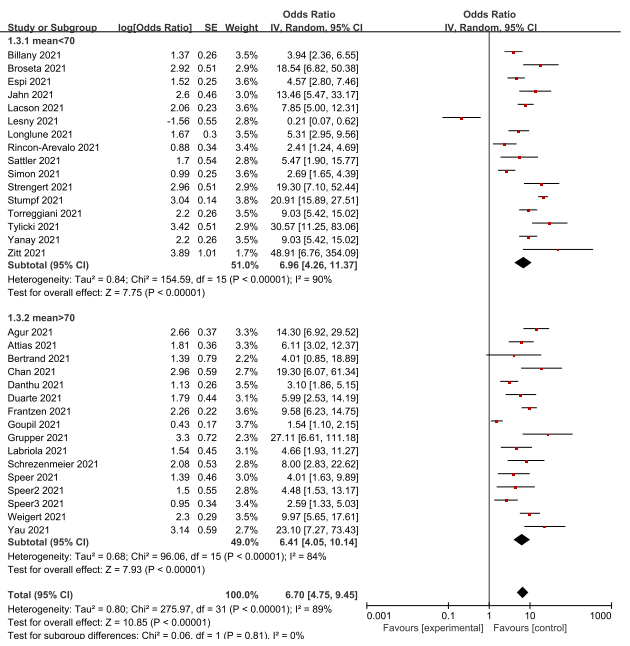


## FigureS4: Subgroup analysis by dialysis vintage


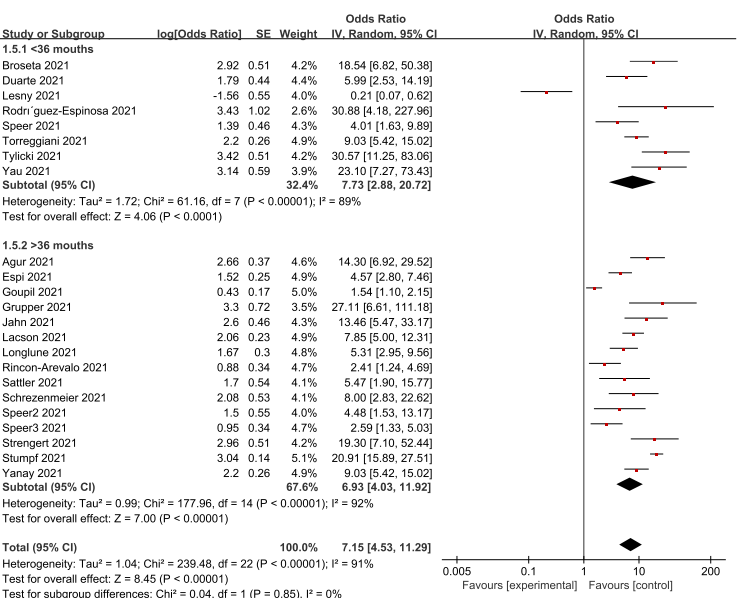


## Figure S5：Subgroup analysis by prevalence of diabetes


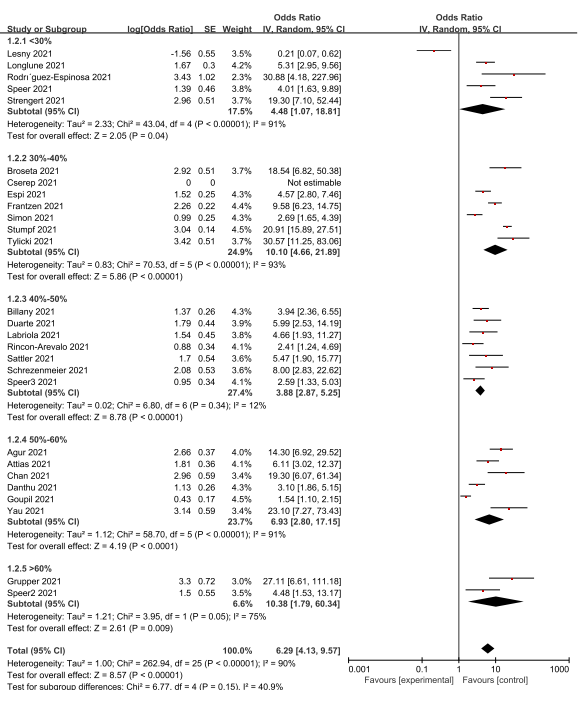


## Figure S6：Subgroup analysis of vaccine types


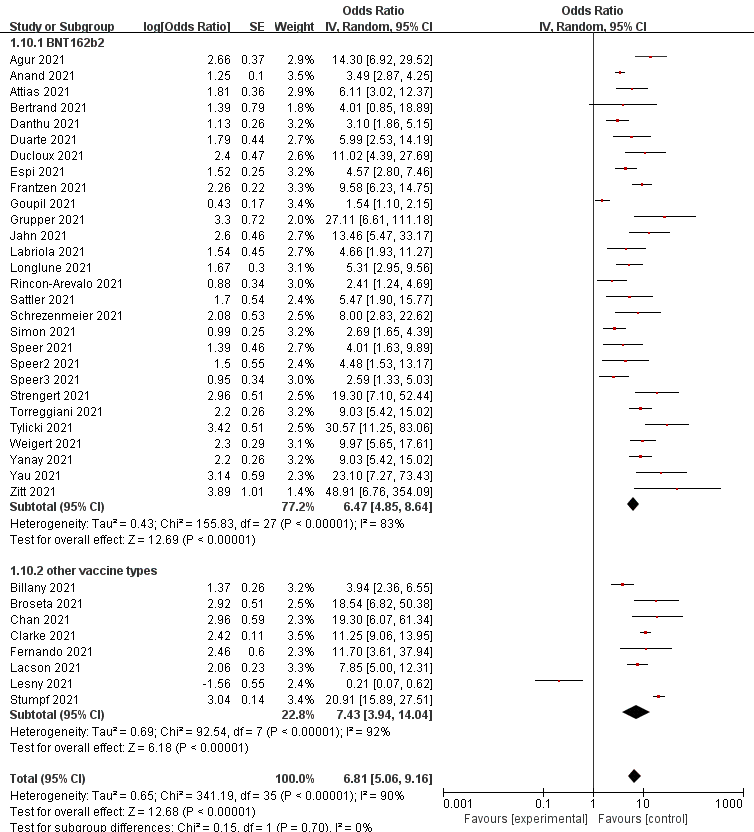


## Figure S7：Subgroup analysis of detected time
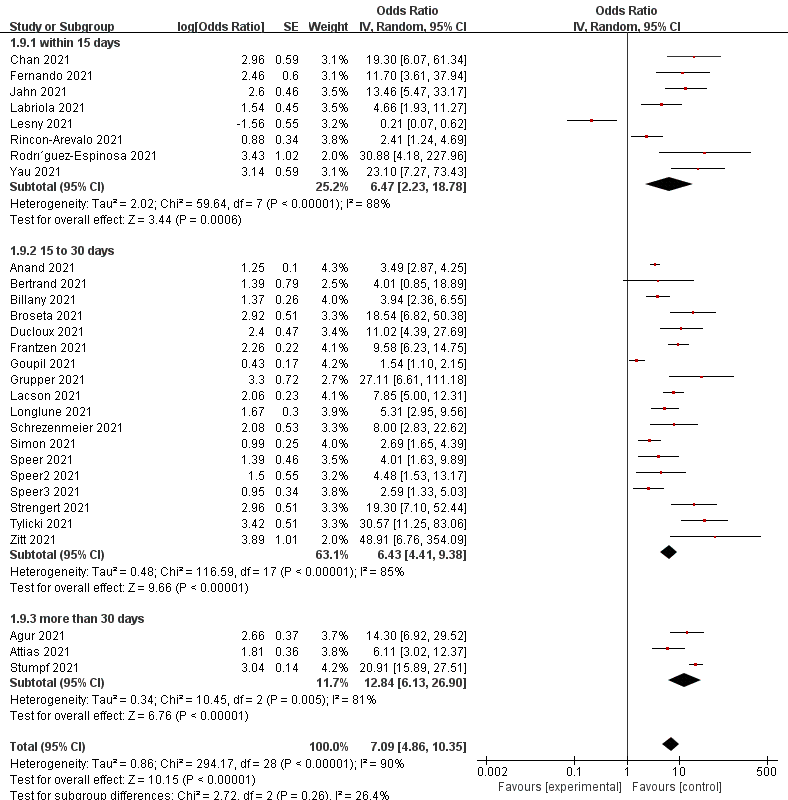


## Figure S8：Subgroup analysis of continents


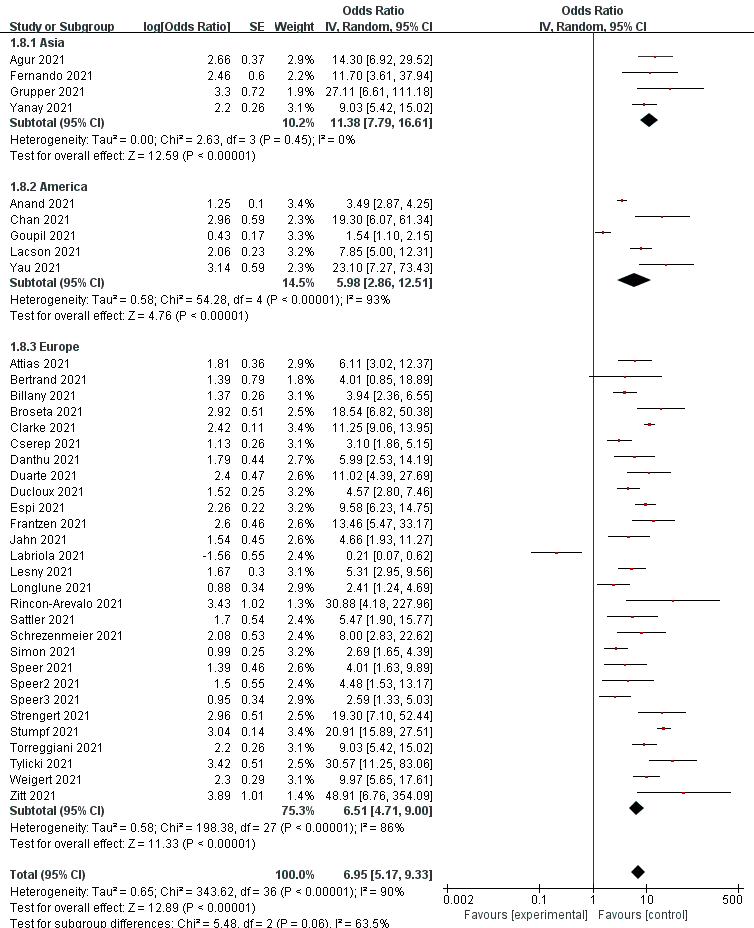


1. **Figure S9: Forest plot of the pain at the injection site after receiving COVID-19 vaccine.**


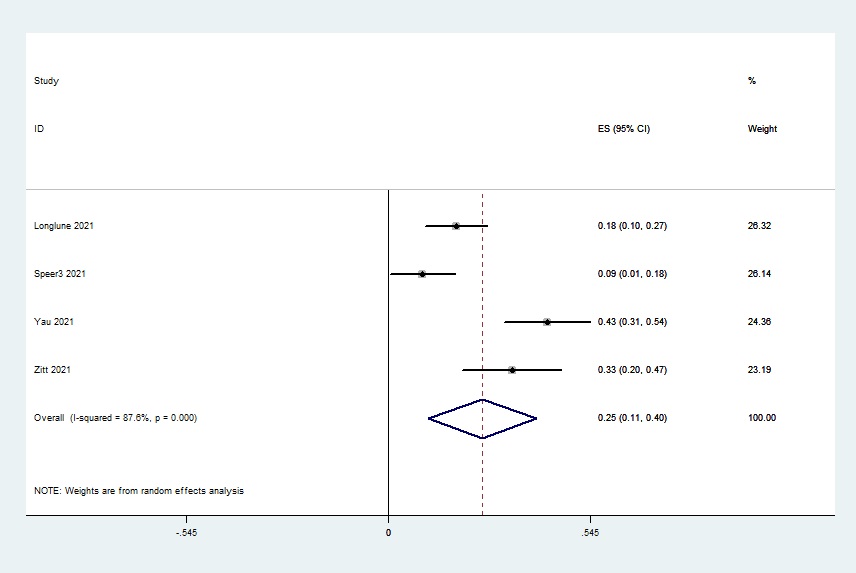


1. **Figure S10: Forest plot of the Fatigue after receiving COVID-19 vaccine.**


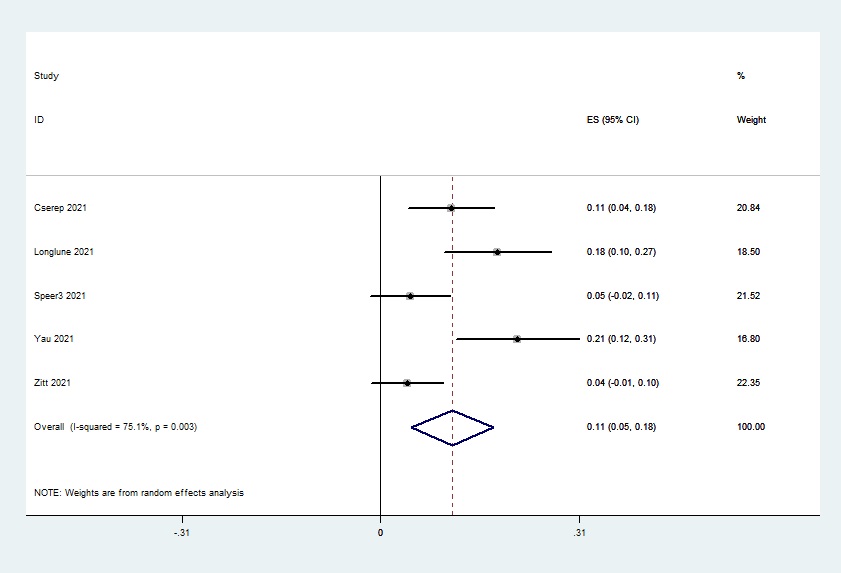

Supplement: Supplementary file 1 [file Data_Sheet_1.docx]
